# Natural Products as Anti-HIV Agents and Role in HIV-Associated Neurocognitive Disorders (HAND): A Brief Overview

**DOI:** 10.3389/fmicb.2015.01444

**Published:** 2016-01-12

**Authors:** Kesava Rao V. Kurapati, Venkata S. Atluri, Thangavel Samikkannu, Gabriella Garcia, Madhavan P. N. Nair

**Affiliations:** Department of Immunology, Institute of NeuroImmune Pharmacology, Herbert Wertheim College of Medicine, Florida International University, MiamiFL, USA

**Keywords:** HIV, neurocognitive disorders, natural products, anti-HIV agents

## Abstract

As the threat of Human Immunodeficiency Virus (HIV)/Acquired Immunodeficiency Syndrome (AIDS) persists to rise, effective drug treatments are required to treat the infected people. Even though combination antiretroviral therapy (cART) provides stable viral suppression, it is not devoid of undesirable side effects, especially in persons undergoing long-term treatment. The present therapy finds its limitations in the emergence of multidrug resistance and accordingly finding new drugs and novel targets is the need of the hour to treat the infected persons and further to attack HIV reservoirs in the body like brain, lymph nodes to achieve the ultimate goal of complete eradication of HIV and AIDS. Natural products such as plant-originated compounds and plant extracts have enormous potential to become drug leads with anti-HIV and neuroprotective activity. Accordingly, many research groups are exploring the biodiversity of the plant kingdom to find new and better anti-HIV drugs with novel mechanisms of action and for HIV-associated neurocognitive disorders (HAND). The basic challenge that still persists is to develop viral replication-targeted therapy using novel anti-HIV compounds with new mode of action, accepted toxicity and less resistance profile. Against this backdrop, the World Health Organization (WHO) suggested the need to evaluate ethno-medicines for the management of HIV/AIDS. Consequently, there is need to evaluate traditional medicine, particularly medicinal plants and other natural products that may yield effective and affordable therapeutic agents. Although there are a good number of reports on traditional uses of plants to treat various diseases, knowledge of herbal remedies used to manage HIV/AIDS and HAND are scanty, vague and not well documented. In this review, plant substances showing a promising action that is anti-HIV and HAND will be explored along with what they interact. Since some plant substances are also known to modulate several cellular factors which are also involved in the replication of HIV and hence their role as potential candidates will be discussed. HIV/AIDS being an exceptional epidemic, demands an exceptional approach and that forms very much focus for the current review.

## Introduction

Since its first discovery in 1981, Human Immunodeficiency Virus (HIV)/Acquired Immunodeficiency Syndrome (AIDS) has killed more than 25 million people worldwide and is today the major threat to human health. However, due to the introduction of highly active antiretroviral therapy (HAART) as well as the impact of preventive measures, the prevalence and incidence of HIV have declined globally over the last decade except for parts of Eastern Europe and Central Asia where a slight increase has been observed (UNAIDS, 2010; Cohen, 2012; Futterman, 2012; Kumari and Singh, 2012; Saxena et al., 2012). Even though HAART provides stable viral suppression, it is not devoid of undesirable side effects, especially in persons undergoing long-term treatment. The present therapy finds its limitations in the emergence of multidrug resistance and accordingly finding new drugs and novel targets is the need of the hour to treat the infected persons and further to attack HIV reservoirs in the body like brain, lymph nodes to achieve the ultimate goal of complete eradication of HIV and AIDS (Vermani and Garg, 2002; Chinsembu and Hedimbi, 2010). Nature has always been considered a basis for providing resources that can be used to fight off infections and treat diseases. Among these resources there have been findings of medicinal plants that have anti-HIV properties with low levels of toxicity. For the management of HIV/AIDS, in 1989 the World Health Organization (WHO) had declared the necessity to assess ethnomedicines and other natural products. To quote: “In this context, there is need to evaluate those elements of traditional medicine, particularly medicinal plants and other natural products that might yield effective and affordable therapeutic agents. This will require a systematic approach” (World Health Organization [WHO], 1989a,b). Moreover, because they are fairly not as expensive and have less side effects, phytomedicines are retrieving patient approval (Vermani and Garg, 2002; Short, 2006). Medicinal effects of plants incline to stabilize physiological function and fix the fundamental cause of the condition (Murray and Pizzorno, 1999). Recently, there has been a sustained bio prospective effort to isolate the active leads from plants and other natural products for treatment of HIV and management of AIDS (Asres et al., 2001; Vermani and Garg, 2002) and also screening of plants based on ethnopharmacological data enhances the ability of discovering novel anti-HIV compounds (Fabricant and Farnsworth, 2001; Farnsworth, 2007). Indigenous knowledge of medicinal plants further provides leads toward therapeutic concept, thereby accelerating drug discovery. Thus, it is essential to explore for new antiretroviral agents which can be combined with or substitute the present resource of drugs against HIV (Klos et al., 2009).

Also, due to the widespread infection of HIV there has been a determination for the discovery and use of medicinal agents that can inhibit the virus. HIV type-1 (HIV-1) displays extraordinary genetic variation and can be phylogenetically classified into three distinct groups and several subgroups (A–K) across the globe. While HIV-1 clade C dominates the HIV-1 epidemic worldwide with more than 50% of the total viral infections, predominantly in Southern Africa, India, and other parts of Asia, HIV-1 subtype B is mainly found in (Western) Europe and in the Americas, accounting for about 10% of the total infections (Rambaut et al., 2001; Hemelaar et al., 2011; UNAIDS, 2011; Hemelaar, 2012; Reddy et al., 2012). In places like Brazil, clade C viruses have been spreading rapidly to replace the previously dominant clade B epidemic (Buonaguro et al., 2007; Gandhi et al., 2009; de Oliveira et al., 2010). The virus basically utilizes the host’s cellular machinery for its own replication leading to extensive viral and host interactions during its life cycle (Kitchen et al., 2001; Lawn et al., 2001; Valenti, 2001; An et al., 2004; Resh, 2005; Broder, 2009; Gandhi et al., 2010). Some of the cellular factors aid replication while others are inhibitory (Balzarini, 1994; Mohri et al., 2001; Benito et al., 2004; Petrovas et al., 2006; Appay et al., 2007; Samikkannu et al., 2010). All these mechanisms lead to chronic immune activation, the key factor in HIV-1 immunopathogenesis. The disease progression (Clerici et al., 1996; Rodriguez et al., 2006; Schader and Wainberg, 2011; Wayengera, 2011) is associated with the gradual T lymphocyte decline, first taking toll of helper CD4^+^ T cells and then the cytotoxic CD8^+^ T cells. The pronounced decline of CD4^+^ T cells occurs due to the destruction of both infected and uninfected cells mainly because of apoptosis of activated cells, diminished hematopoiesis and thymic maturation. Apart from inducing immune dysfunction, HIV-1 infection affects neuronal function by significantly increasing several cellular proteins in the brain, including amyloid precursor protein (APP), especially in the axons present in the subcortical white matter tracts (Esiri et al., 1998; Green et al., 2005; Soontornniyomkij et al., 2012). During HAART therapy HIV persists in the brain and local inflammatory responses to the virus can lead to higher APP production and β-amyloid deposition which may lead to the progression of Alzheimer’s disease and HAND (Giunta et al., 2011). Accordingly, finding new drugs and novel targets is the need of the hour to treat the infected persons and further to attack HIV reservoirs in the brain to achieve the ultimate goal of complete eradication of HIV and AIDS.

Extensive attention has been given to the pathogenesis of HIV-1 infection and associated immunologic mechanisms, however, the basic challenge that still persists is to develop viral replication-targeted therapy using novel anti-HIV compounds with new mode of action, accepted toxicity, and less resistance profile. Accordingly, in this review, plant substances showing a promising anti-HIV activity and HAND will be summarized along with the targets they interact including the specific objectives for the pursuit of plants and other natural products with identified active substances and mechanisms as well as those with unidentified active mechanisms against HIV, AIDS, and other neurocognitive disorders.

## HIV-Associated Neurocognitive Disorders (HAND) and Antiretroviral Therapy (ART)

Individuals infected with HIV have conditions of fluctuating degrees of impairment of cognition and associated functioning and can develop HIV-associated neurocognitive disorders (HAND; Antinori et al., 2007). Invasion and replication of HIV in the brain parenchyma is accomplished by brain perivascular macrophages, endogenous microglia, and some astrocytes that are infected and can initiate the neuropathogenesis of HAND (Kaul, 2009; McArthur et al., 2010). Immune activation of resident glia and neuroinflammation are associated with this infection and neuronal injury. The general occurrence of HAND and related diseases continue to be at high levels even with the extensive use of antiretroviral therapy (ART) that has radically reduced the occurrence of the severest form of HAND, HIV-associated dementia (HAD; Sacktor et al., 2002; McArthur et al., 2004; Nath et al., 2008; Heaton et al., 2010). The persistence of this high risk for HAND in individuals experiencing effective control of systemic HIV viral load is incompletely explained, and suggested factors include effects of aging on brain vulnerability, persistence of HIV replication in brain macrophages, evolution of highly neurovirulent CNS HIV strains, and even long-term CNS toxicity of ART (Heaton et al., 2010, 2011). The features linked with increasing the inflammatory setting within the CNS and HIV replication are what drive the pathogenesis of HAND. Neuronal damage associated with HAND can be augmented by subordinate effects of aging, movement of activated monocytes, systemic immune activation and drug abuse and can continue in spite of the effective systemic control of HIV replication by ART (Gannon et al., 2013). Active complementary neuroprotectants are substances that can overturn systemic immune activation and associated inflammation both systemically and within the CNS (Kaul and Lipton, 2006; Kraft-Terry et al., 2009; Grovit-Ferbas and Harris-White, 2010; McArthur et al., 2010). Drugs that aim toward these cellular pathways could quickly ease testing and application of possible complementary neuroprotective approaches against HAND. Prevention and treatment of HAND requires strategies aimed at suppressing CNS HIV replication and effects of systemic and CNS inflammation in aging and substance-abusing HIV populations. Use of improved CNS-penetrating ART must be accompanied by evaluation of potential ART neurotoxicity.

## Factors and Neurotoxic Compounds Inducing HIV-Associated Neurocognitive Disorders

After initial infection and seroconversion, HIV usually reaches the brain. Infected monocytes migrating across the blood–brain barrier (BBB), which can be seen as a “Trojan horse” mechanism, is one concept suggested for potential viral entrance. After these infected monocytes migrate across the endothelium, they settle as infected perivascular macrophages. It has been proposed that the virus is spread by cell-to-cell interaction between microglia cells and macrophages (Kaul et al., 2001). The main cell types that upkeep viral replication in the brain are multinucleated giant cells (fused microglia cells and macrophages) and macrophages in the perivascular spaces (Gonzalez-Scarano and Martin-Garcia, 2005). A multifaceted cascade is prompted within the brain with recurrent exposures and heightened viral replication within microglia. Inside the brain tissue is where viral proteins are released which stimulate astrocytes and microglia to produce cytokines, chemokines, and other inflammatory agents (Nottet and Gendelman, 1995; Achim and Wiley, 1996). Release of viral proteins is further stimulated by these cell-encoded indicators that support the increase of HIV replication in microglia cells. This can produce a toxic effect on neurons, causing neuronal dysfunction and neuronal loss. Nevertheless, when a collection of these factors are existing along with the HIV infection, a distinctive neuropathological signature is seen. Pathological alterations are usually minor with irregularities mostly taking place in basal ganglia, brain stem, central white matter, frontal cortices, thalamus, and eventually leading to HAND (Sharer, 1992). There are studies exploring the associated risk factors for the neurocognitive disorders in HIV patients and found that in some but not in all studies, CD4 nadir count (Munoz-Moreno et al., 2008; Ellis et al., 2011; Heaton et al., 2011) and detectable HIV plasma viral load (Tozzi et al., 1999; Chang et al., 2002; Devlin et al., 2012), have been associated with neurocognitive impairment (Gonzalez et al., 2003; Paul et al., 2005; Vitiello et al., 2007; Ettenhofer et al., 2009; Simioni et al., 2010). Shortening in telomere length (TL), which has been used as a biomarker of aging, has also been reported in individuals with an HIV+ status (Zanet et al., 2012; Malan-Muller et al., 2013; Pathai et al., 2013). Also, shortened TL reported to increase the neurocognitive impairment and risk of dementia in non-HIV populations (von Zglinicki et al., 2000; Panossian et al., 2003; Honig et al., 2006) which can be a marker for atypical age-related cognitive decline (Martin-Ruiz et al., 2006; Valdes et al., 2008; Yaffe et al., 2009).

## Naturally Derived Anti-HIV Agents

**FIGURE 1:**
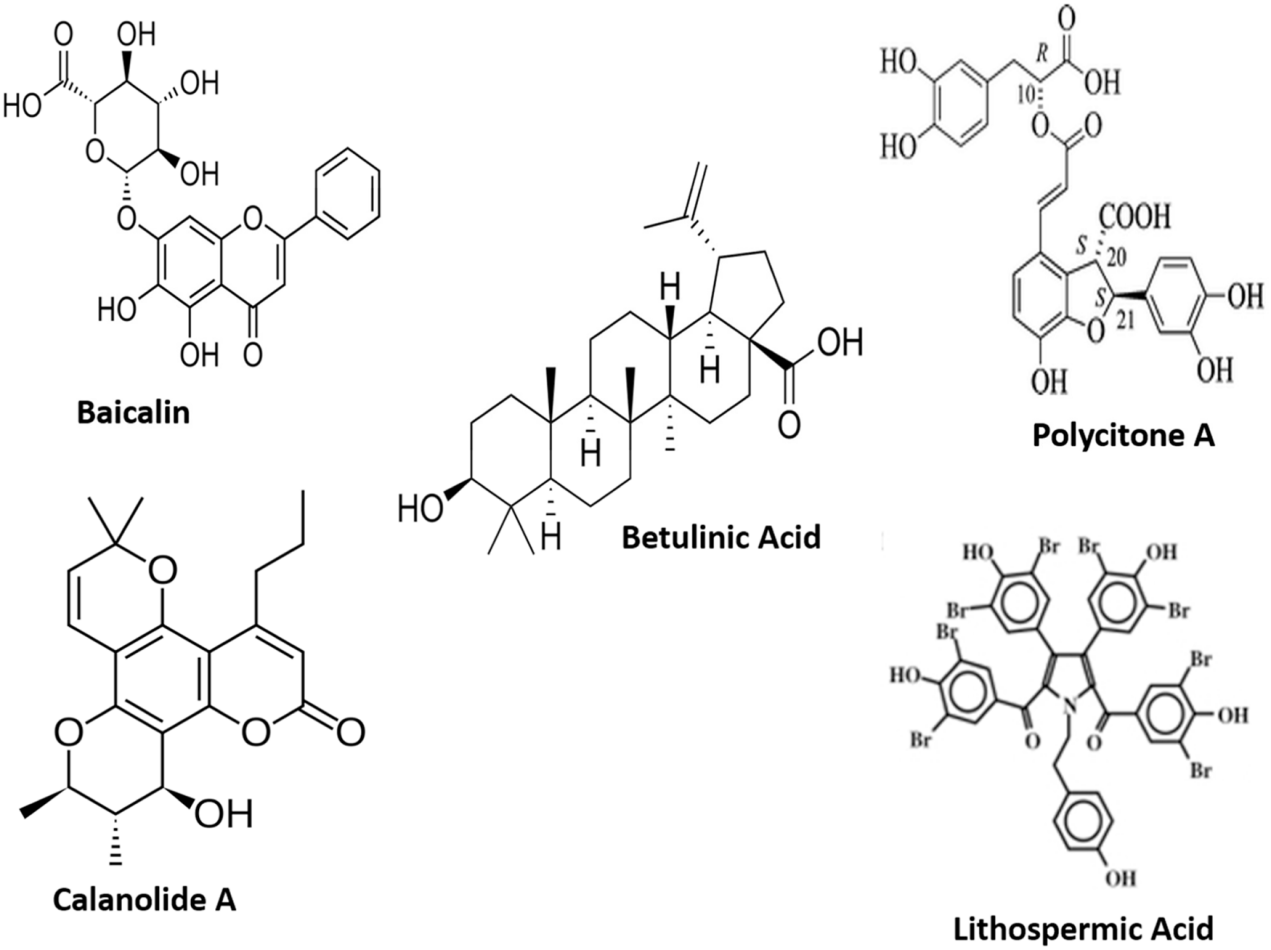
**Structural formulae of representative anti-HIV natural products**.

**FIGURE 2:**
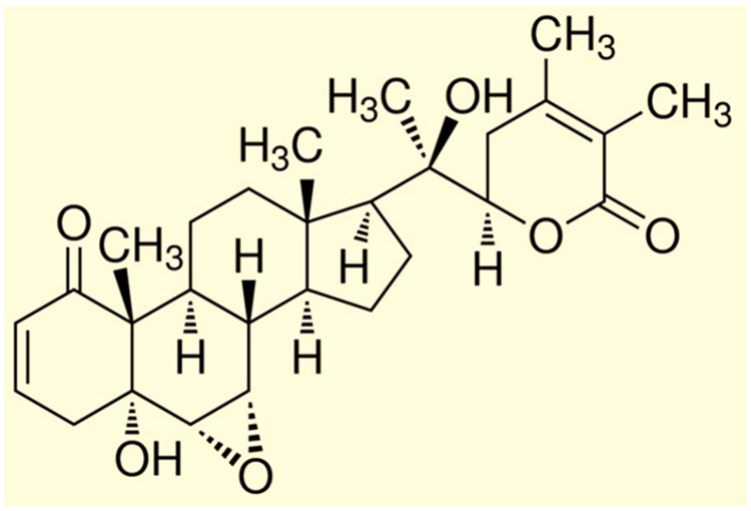
**Chemical structure of Withanolide A.** [Reproduced with permission from Kurapati et al. (2014).]

The following natural products namely Calanolides (Coumarins), Betulinic acid (a Triterpene), Baicalin (a Flavonoid), Polycitone A (an Alkaloid), Lithospermic acid (a Polyphenolic) can be mentioned as promising for anti-HIV agents (**Figure 1**) whereas Withanolides (Steroidal lactones; **Figure 2**) for HIV-associated neurocognitive disorders.

### Coumarins

An example of non-nucleoside specific reverse transcriptase inhibitors (NNRTI) of the virus are calanolides which are a type of coumarin derived from different tropical tree species of the *Calophyllum* (Clusiaceae family; Dharmaratne et al., 2002). Calanolide A (**Figure 1**), (Vlietinck et al., 1998), Calanolide B and its derivative 7,8-dihydrocalanolide B can prevent cytopathogenic effects of HIV-1 on host cells and are attained from the *Calophyllum lanigerum.* Cordatolide A and B are similar in structure to Calanolides and can inhibit replication of HIV-1. These compounds are extracted from the *Calophyllum cordato-oblongum* (Lee et al., 1994). Suksdorfin (Yu et al., 2003) is another compound that also has inhibitory effects on replication of the virus in the T cell line and is a pyrocoumarin derivative that can be extracted from the *Angelica morii* and *Lomatium suksdorfii* fruits from the Apiaceae family (Zhou et al., 2000).

### Terpenes

Antiretroviral activity with diverse mechanisms of action have been observed with some triterpenoids. Betulinic acid (**Figure 1**), platanic and oleanolic acid tested in H9 lymphocyte cells have shown inhibition of HIV and can be obtained from the leaves of *Syzigium claviflorum* (Min et al., 1999). Inhibition of HIV-1 replication in these cells were observed with oleanolic acid which can be attained from methanolic extract of the *Xanthoceras sorbifolia*, wood from the Sapindaceae family (Kashiwada et al., 1998). Potent inhibitory activity against HIV-1 protease has been seen with maslinic acid derived from the *Geum japonicum* (Xu et al., 1996). Anti-HIV replication activity in H9 cells is observed with Celasdin B that is derived from ethanolic extract of the *Celastrus hindsii* which is from the Celastraceae family (Yao-Haur and Li-Ming Yang, 1997). The protostanes, garcisaterpenes A and C can suppress HIV-1 RTase activity and are obtained from the ethyl acetate extract of the stems and bark of *Garcinia speciosa* (Rukachaisirikul et al., 2003). Lanostane-type triterpene has also shown inhibition of HIV replication in H9 cells and is a suberosol obtained from ethanolic extract of the leaves and stems of *Polyalthia suberosa* from the Annonaceae family (Li et al., 1993). Triterpene lactone, lancilactone C extracted from the roots and stems of *Kadsura lancilimba* is another compound that restrains HIV replication in these cells (Chen et al., 1999). The 12-*O*-tetradecanoylphorbol-13-acetate (TPA), a phorbol diester, can inhibit cytopathogenic effects of HIV-1 and is attained from methanolic extract of *Croton tiglium* from the Euphorbiaceae family. Prostratin, a phorbol ester also has anti-HIV properties and is extracted from *Homalanthus nutans* from the Euphorbiaceaen family (El-Mekkawy et al., 2000).

### Flavonoids

Favorable anti-HIV activity have been exhibited by flavonoids and associated polyphenols. They are known for having antioxidant properties and have been found to show antiviral activity in different cell cultures (Orhan et al., 2010). Baicalin (**Figure 1**) inhibits HIV replication in PBMC in a dose dependent fashion and is an anti-HIV flavonoid extracted from *Scutellaria baicalensis* (Ohtake et al., 2004). The 6,8-diprenylaromadendrin and 6,8-diprenylkaempferol, prenylated flavonoids, also show anti-HIV activity in the XTT-based, whole-cell screen and are derived from the extract of *Monotes africanus* (Meragelman et al., 2001). Flavonoid gallate ester and quercetin 3-*O*-(2-galloyl) *a*-L-arbinopyranose can inhibit integrase activity of HIV-1 and are obtained from ethanolic extract of *Acer okamotoanum* from the Aceraceae family (Kim et al., 1998). Hinokiflavone, robustaflavone, and biflavonoids have demonstrated inhibition of the polymerase of HIV-1 reverse transcriptase (RT) and are attained from methanolic extracts of leaves and twigs of *Rhus succedanea* from the Anacardiaceae family (Lin et al., 1997). Wikstrol B, a biflavonoid, also shows anti-HIV activity and can be isolated from extracts of roots of *Wikstroemia indica* from the Thymelaeaceae family (Hu et al., 2000). Xanthohumol, a prenylchalcone that has demonstrated inhibition of HIV-1 and is extracted from hops *Humulus lupulus* (Wang et al., 2004).

### Alkaloids

Different types of alkaloids have shown anti-HIV activity. One of the natural products with interesting activity on RT is polycitone A (**Figure 1**), an aromatic alkaloid isolated from the marine ascidian *Polycitor* sp. Polycitone A exhibits potent inhibitory activity on both RNA- and DNA-directed DNA polymerases (Loya et al., 1999). Papaverine, an alkaloid can inhibit HIV replication and is extracted from *Papaver sominiferum* from the Papaveraceae family. Buchapine is a type of quinolone that has shown inhibition of cytopathogenic effects of HIV-1 and is isolated from *Eodia roxburghiana* (McCormick et al., 1996). Nitidine also shows anti-HIV activity and is extracted from the roots of *Toddalia asiatica* of the Rutaceae family (Tan et al., 1991). A piperidine flavone related alkaloid *O*-demethylbuchenavianine shows a hindrance to the activity of HIV and is attained from *Buchenavia capitata* of the Combretaceae family (Beutler et al., 1992). Harmine has shown inhibition of HIV replication in H9 cells and is derived from *Symplocos setchuensis* (Ishida et al., 2001). 1-Methoxy canthionone has anti-HIV properties and is obtained from *Leitneria floridana* (Xu et al., 2000). Hypoglaumine B, Troponine B, and Troponine A are sesquiterpene pyridine alkaloids that have shown to also possess anti-HIV properties and are isolated from *Tripterygium wilfordii* and *Tripterygium hypoglaucum* (Duan et al., 2000).

### Phenolics

Because of heightened phytohaemagglutinin-induced lymphocytes proliferation, prolonged administration of polyphenol-rich fruit juices is believed to be promising to HIV-positive individuals. There are several tannins and related phenolic substances which show virucidal effects in several viral systems. Lithospermic acid (**Figure 1**) isolated from *Salvia miltiorrhiza* has strong anti-HIV activity in H9 cells (Abd-Elazem et al., 2002). Punicalagin, chebulagic acid, and punicalin are hydrolysable tannins that demonstrate anti-HIV activity and come from *Terminalia chebula* (Lim et al., 1997). Repandusinic acid has shown inhibition of HIV-1 RTase and is extracted from *Phyllanthus niruri* of the Euphorbiaceae family (Ogata et al., 1992). Monopotassium and monosodium salts of isomeric caffeic acid tetramer have shown inhibition of HIV replication and are obtained from the aqueous acetone extract of *Arnebia euchroma* of the Boraginaceae family (Kashiwada et al., 1995). *Camellia*-tannin H shows inhibition of the HIV-1 protease and is extracted from the pericarp of *Camellia japonica* (Fuller et al., 1999). Galloyl glucoses and gallic acid exhibited a hindrance of HIV integrase and are extracted from *Terminalia chebula* of the Combretaceae family (Ahn et al., 2002; Park et al., 2002). Mallotojaponin is a dimeric phloroglucinol derivative that inhibits activity of HIV-1 RTase and is extracted from the pericarps of *Mallotus japonicus*. The curcuminoids have shown inhibition of HIV-1 and HIV-2 protease and are extracted from rhizomes of *Curcuma longa* (Roth et al., 1998). Peltatol A is a prenylated catechol dimer that shows anti-HIV activity and is extracted from *Pothomorphe peltata* of the Piperaceae family (Gustafson et al., 1992).

### Lignans

Several lignans have antiviral properties (Charlton, 1998). Phyllamyricin B and its lactone retrojusticidin B display inhibition of HIV-RTase activity and are obtained from chloroform extract of *Phyllanthus myrtifolius*/*P. urinaria* of the Euphorbiaceae famly (Liu and Li, 1995). Anolignan B, anolignan A, and dibenzylbutadiene lignans have shown inhibition of HIV-1 RTase and are extracted from *Anogeissus acuminata* (Rimando et al., 1994). Gomisin has been found to be one of the strongest inhibitors of HIV replication and is obtained from *Kadsura interior* (Chen et al., 1997).

### Quinones

Plumbagin, 1,4-naphthoquinone, juglone, and vitamin K3 are naphthoquinones that all demonstrate HIV inhibitory activity (Min et al., 2002). Conocurvone is a trimeric naphthoquinone that shows effective anti-HIV activity and is extracted from *Conospermum incurvum* of the Proteaceae family (Decosterd et al., 1993).

### Saponins

Actein is a tetracyclic triterpenoid saponin that exhibits strong anti-HIV activity and derives from the rhizome of *Cimicifuga racemosa* (black cohosh), (Sakurai et al., 2004).

### Xanthones

Swertifrancheside is a flavonone–xanthone glucoside that has shown inhibition of HIV-1 RTase and is extracted from *Swertia franchetiana* (Wang et al., 1994). Macluraxanthone B is a prenylated xanthone that also shows anti-HIV activity and is extracted from *Maclura tinctoria* of the Moraceae family (Groweiss et al., 2000).

**Table 1** list the ongoing or completed clinical trials of anti-HIV effect of natural substances.

**Table 1:** Summary of clinical trials of natural products for treating HIV and AIDS.

Natural product and Country	Design	Aim	Treatment plan	Study phase and Reference
*Echinacea purpurea*- Spain	Non-Randomized, Single Group Assignment two arms, Open Label trial	To characterize drug interactions between medicinal herb *Echinacea purpurea* widely used by HIV infected patients and its interaction with protease inhibitor Darunavir/Ritonavir	Recruiting 15 HIV-1 infected patients on antiretroviral therapy with darunavir/ritonavir 600/100 mg twice a day for 4 weeks. Treating these patients with *Echinacea purpurea* for 14 days, 500 mg every 6 h.	Phase 4 (Molto, 2010)
*Echinacea purpurea*- Spain	Non-Randomized, Single Group Assignment two arms, Open Label trial	To characterize drug interactions between one medicinal herb: *Echinacea purpurea* and the protease inhibitor etravirine	Recruiting 15 HIV-infected patients on stable antiretroviral therapy including etravirine at the dosage of 200 mg twice daily during at least 4 weeks. Treating these patients with *Echinacea purpurea* root (500 mg every 8 h) in addition to their antiretroviral treatment, from day 1 until 2 weeks later (day 14).	Phase 1 (Fundacio Lluita Contra la SIDA, 2011)
*Triptolide woldifiion*- China	Randomized, Factorial Assignment, two arms, Open Label trial	To evaluate the effects and side effects of ARV treatment in acute HIV-1 infection phase, and to evaluate the impact of Triptolide wilfordii on HIV-1 reservoir	Recruiting 18 naive-treatment patients with acute HIV-1 infection phase, and treating with the antiretroviral drugs (Tenofovir plus lamivudine plus Lopinavir/ritonavir plus raltegravir). Six months later, treating these 12 patients with Triplitode.	Phase 3 (Li, 2014)
*Triptolide wilfordii*- China	Randomized, Crossover Assignment two arms, Double Blind, placebo-controlled trial	To evaluate impact of Triptolide wilfordii on T-cell immune activation and inflammation biomarkers in HIV-infected immunological non-responders	Recruiting 120 patients from 4 HIV/AIDS clinical centers and treating the invention group with *Triptolide wilfordii* (20 mg tid po) for 24 weeks.	Phase 1 (Lu, 2013) Phase 2
*Tripterygium wilfordii* Hook F (TwHF)- China	Parallel, Non-Randomized, two arms, open label trial	Safety and Efficacy Study of *Tripterygium wilfordii* Hook F Extract in cART-Treated HIV Patients With Poor Immune Responses	Recruiting 23 patients immunological non-responders, 19 receive TwHF extract (10 mg tid po) plus current cART, while four patients continue their current cART for 12 months.	(Murray et al., 2010; Piconi et al., 2011)
*Moringa oleifera*- Zimbabwe	Observational, Case-Crossover, Prospective study	To study the effect of simultaneous treatment with antiretroviral drugs (nevirapine and efavirenz) and the herb Moringa	Recruiting 19 HIV-positive patients to compare the steady-state pharmacokinetics of nevirapine and efavirenz before and after supplementation with *Moringa oleifera* leaf powder.	(Sebit et al., 2000)
Immunity 1 (Fuzheng 1)- China	Multi-center, Randomized, two arms, Double-blind, Placebo-controlled Clinical Trials	Study the effect of Immunity 1 (Fuzheng 1) on Immune Reconstitution of HIV Patients	Recruiting 60 HIV-positive patients (WHO clinical stage I and II) and treating with Fuzheng 1 (8.75 g twice a day) half an hour before breakfast and dinner, mixing with water, for six successive cycles of 30 days.	(Wang, 2009a)
Immunity 1 and 3 (Fuzheng 1 and 3): Composed of herbs which have tonic and detoxific function- China	Multi-center, Randomized, three arms, Double Blind, placebo-controlled trial	To evaluate the efficacy and safety of combination of Traditional Chinese Medicine (TCM) and Highly Active Antiretroviral Therapy (HAART) on immune reconstitution of HIV/AIDS patients	Recruiting 180 HIV infected patients and treating with Fuzheng 1 or 3 (8.75 g/twice a day) half an hour before breakfast and dinner, mixing with water, for six successive cycles of 30 days.	(Wang, 2009c)
Immunity 2 (Fuzheng 2)- China	Parallel, randomized, two arms, Double Blind, placebo-controlled trial	To evaluate the effect of immune 2 (Fuzheng 2) on immune reconstitution of adult HIV/AIDS patients who have received HAART	Recruiting 180 HIV infected patients and treating with Fuzheng 2 (6.25 g twice a day) half an hour before breakfast and dinner, mixing with water, for six successive cycles of 30 days.	(Wang, 2009b)

### Ameliorating Effects of *Withania somnifera* (Ashwagandha) Root Extract on HIV-Associated Neurocognitive Disorders

It is well known that use of alcohol and drugs of abuse are associated with increased risk of attaining HIV-1 infection as well as augmented disease progression (Nair et al., 2004, 2009; Dhillon et al., 2007; Atluri et al., 2014). Further, studies have shown that use of alcohol and drugs of abuse are also risk factors for dementia and other cognitive disorders (Brandt et al., 1983; Darke et al., 2000; Atluri et al., 2015; Hidalgo et al., 2015; Roy et al., 2015; Sagar et al., 2015; Samikkannu et al., 2015a,b). Along this line, impacting more than 15 million people worldwide, Alzheimer’s disease (AD) is the most frequent form of senile dementia (Kim et al., 2011). This number will surely increase quickly in the future with augmented life expectancy. The pathological hallmarks of AD are complex and include neuronal degeneration, neuro-inflammation, toxic β-amyloid (AB) plaques, abnormal neurofibrillary tangles, and a decline of neurochemicals which are essential for neuronal transmission (McArthur et al., 2003, 2010; Robertson et al., 2007; Heaton et al., 2010, 2011). The precise mechanisms of β-amyloid that lead to neurotoxicity continue to be a question but its cytotoxicity to neuronal cells has been acknowledged as one of the main features in AD pathology (Sacktor et al., 2001). In AB-induced cytotoxicity it seems that some of the possible mechanisms involved are oxidative stress and free radical formation (Xu and Ikezu, 2009). In HIV infection, neuronal degeneration is also a main feature. In AIDS patients, a noteworthy increase in brain APP has been observed especially in the axons found in the subcortical white matter tracts (Esiri et al., 1998; Green et al., 2005; Anthony et al., 2006; Lindl et al., 2010). HIV can persist in the brain during HAART therapy and local inflammatory responses can cause an increase of APP production and exposure to amyloid deposition (Anthony and Bell, 2008). During HIV induced neuronal degeneration and development of Alzheimer’s disease, β- amyloid buildup may be used as an indicator of early neuronal degeneration.

**FIGURE 3:**
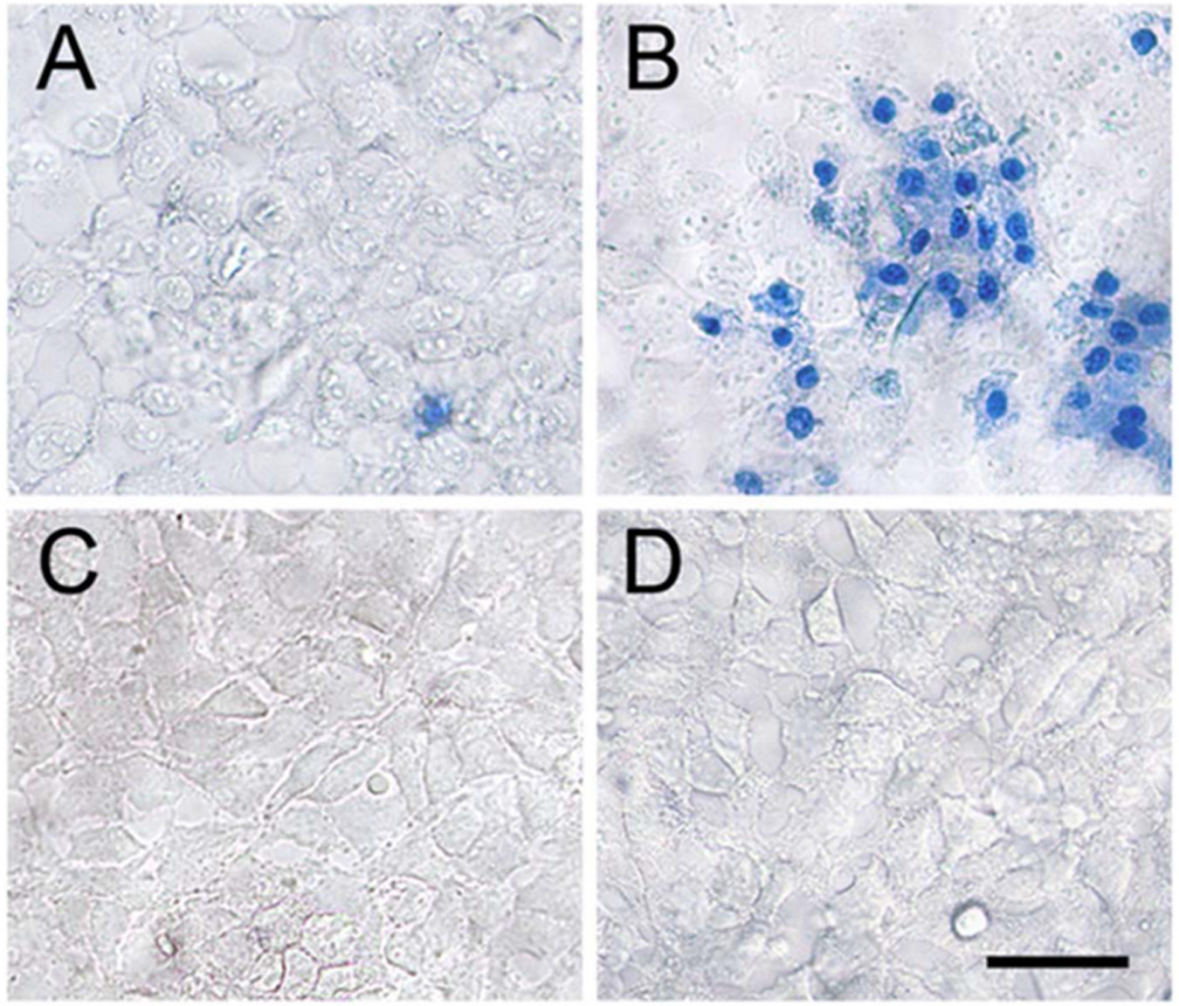
**Effect of ASH in reversal of β-Amyloid induced cell death.** Treating the SK-N-MC cells with β-Amyloid **(B)** resulted in significant cell death when compared to the untreated control cells **(A)**. Treating the cells with ASH **(C)** and ASH plus β-Amyloid **(D)** exhibited good viability as evidenced by exclusion of trypan blue dye. [Reproduced with permission from (Kurapati et al., 2014).]

**FIGURE 4:**
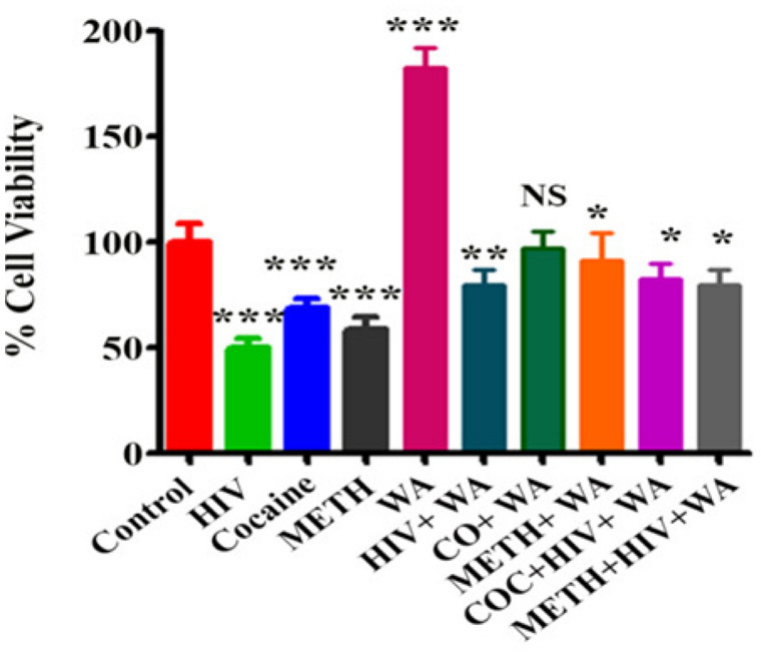
**Effect of Withanolide A on viability of SK-N-MC cells treated with HIV-1Ba-L (clade B), Cocaine and Methamphetamine (METH): SK-N-MC cells were infected with HIV in the presence of Cocaine/METH.** The MTT assay is showing that Withanolide A reverse the neurotoxic effects on cell viability induced by HIV, Cocaine, METH and combined treatment. Withanolide A alone showed significant increase in cell viability. The values were expressed as percentage of cell viability compared to control cells and are the mean ± SD of four experiments. ^∗∗∗^, ^∗∗^, ^∗^ indicates a statistically significant difference (*p*,0.0001), (*p*,0.0006), and (*p*,0.0185), respectively, compared to controls. WA, Withanolide A; CO, COC, Cocaine; METH, Methamphetamine. [Reproduced with permission from Kurapati et al. (2014).]

A medicinal plant that has many therapeutic properties such as antioxidant and immunomodulatory properties, memory enhancer, nerve tonic and antistress is *Withania somnifera* (L.) Dunal, also known as ‘ashwagandha’ (ASH) or ‘Indian ginseng’ and has had a significant upsurge in pharmacological studies in the recent years (Mishra et al., 2000; Kulkarni and Dhir, 2008). In cultured neurons and in rodents injected with Aβ 25–35, neurite outgrowth has been induced by withanoside IV and withanolide A extracted from the roots (Kuboyama et al., 2005). Root extracts from this species have also been shown to significantly reduce the number of hippocampal degenerating cells in the brains of stressed rodents (Jain et al., 2001) and were neuro-protective in animal models of Parkinson’s disease (Sankar et al., 2007). Accumulation of oligomers and β-amyloid peptides (Aβ), behavioral deficits and plaque pathology have been upturned in the brains of old and middle-aged APP/PS1 Alzheimer’s disease transgenic mice with the oral dispensation of a semi-purified extract of the root of *W. somnifera* mainly involving withanosides and withanolides (Sehgal et al., 2012). However, there is a scarcity of data on the mechanisms related to the possible defensive effects of *W. somnifera* root against HIV-1Ba-L (clade B) infection and β-amyloid (1–42)-induced cytotoxicity. In a confocal microscopic analysis, β-amyloid treated SK-N-MC cells (neuronal cell line) showed a reduction in spine area, dendrite diameter, spine density, total dendrite, and a loss of spines when compared to untreated cells (Kurapati et al., 2013). Nonetheless, the toxic effects were counteracted when ASH root extract was added to β-amyloid treated cells (Kurapati et al., 2013). Further, as demonstrated by increased trypan blue stained cells, β-amyloid prompted cytotoxic effects in SK-N-MC cells. Nevertheless, the toxic effects were neutralized when ASH root extract was added to β-amyloid treated cells (**Figure 3**), (Kurapati et al., 2014). This observation was supported by the levels of acetylcholinesterase activity, cellular localization of β-amyloid, and MTT formazan exocytosis, confirming the defensive effects of ASH root extract against β-amyloid prompted toxicity (Kurapati et al., 2013, 2014). Also, Withanolide A (WA; **Figure 2**) demonstrated the same pattern using MTT assay as a parameter and is a refined component of ASH (**Figure 4**) (Kurapati et al., 2014). These observations suggests that neuroprotective properties of ASH root extract may offer some clarification for the ethnopharmacological usages of ASH in traditional medicine for cognitive and other HIV associated neurodegenerative disorders and additionally ASH could be a possible new drug to reduce the brain amyloid load and/or improve the HIV-1 associated neurocognitive damages.

Apart from our studies on ASH, reports are scanty on the effect of natural compounds for the treatment of HIV associated neurocognitive disorders. *Ginkgo biloba* has been known as a traditional medicine for people with dementia. Zou et al. (2007) reported that administration of EGb 761, a standardized formulation of *Ginkgo biloba* extract, reported to protect Tat transgenic mice from Tat-induced neuro toxic effects like developmental retardation, inflammation, death, astrocytosis, and neuron loss by down-regulating the glial fibrillary acidic protein (GFAP) expression. In GFAP null Tat-transgenic mice model, reduced Tat-induced neuropathological phenotypes including macrophage/microglia activation, central nervous system infiltration of T lymphocytes, and oxidative has been reported.

*Hypericum perforatum* (St John’s Wort) extracts are generally used for the treatment of mild to moderately severe depression. Role of P27^SJ^_,_ a protein present in a laboratory callus culture of *Hypericum perforatum* was explored to control HIV transcription and replication in microglia and astrocytes (Darbinian-Sarkissian et al., 2006). This study reported that P27^SJ^ suppress transcription of the HIV-1 Genome/viral replication (50%) by interacting, inhibiting the DNA-binding activity and altering subcellular localization of CCAAT/enhancer-binding protein beta (C/EBPb), a transcription factor that regulates expression of the HIV-1 genome. Also, P27^SJ^ reported to interact with HIV-1 Tat and inhibits its activity on transcription of HIV-1 LTR and relocalizing it to the cytoplasm. Therefore P27^SJ^ is a potential therapeutic agent for inhibiting the HIV-1 induced neurotoxicity in the brain by suppressing its replication and inhibiting inflammatory response induced by proinflammatory Tat-inducible chemokines such as MCP-1. Few studies reported the potential adverse St. John’s Wort extracts drug reactions and interactions especially with other antidepressants, with coumarin-type anticoagulants, the immunosuppressants cyclosporine and tacrolimus, protease and RT inhibitors used in anti-HIV treatment (Schulz, 2006).

For inhibiting the HIV replication in the brain or nullifying the neurotoxicity induced by HIV replication and its proteins, further studies are necessary to explore the neuroprotective effect of natural compounds like Sage (*Salvia officinalis, Salvia lavandulaefolia, Salvia lavandulifolia*), Bacopa (*Bacopa monnieri*), Gotu kola (*Centella asiatica*), which are being used as a therapeutic alternative medicines for memory and cognitive improvement.

## Plant Compounds Taking Action on Viral Marks and Advantages

In the study of plant extracted anti-HIV compounds there have been significant improvements that are centered on their activity *in vitro* and the mechanisms that are used. Some of these compounds will be discussed in this review along with the viral targets they interact with. Covering all the natural substances that possess these anti-HIV properties is still a work in progress. Most of these compounds interfere with early steps in the HIV replication, such as the virus entry steps and on the viral enzymes RT and integrase.

### Virus Entry

Many protein interactions are involved in HIV entry and have the potential to become targets for the development of new entry blockers. The HIV fusion inhibitor, Enfuvirtide (Jiang et al., 2002) does not have oral bioavailability but requires two injections per day and can cause significant local side effects. Compounds that are recognized as strong inhibitors of HIV-1 and HIV-2 replication *in vitro* are sulfated polysaccharides (Witvrouw and De Clercq, 1997; Lüscher-Mattli, 2000). These compounds exert their anti-HIV properties by shielding off the positively charged amino acids in the V3 loop of the glycoprotein gp120 from the viral envelope of HIV (Witvrouw and De Clercq, 1997). The V3 loop is necessary for virus attachment to cell surface heparan sulfate, a primary binding site, before more specific binding occurs to the CD4 receptor of CD4^+^ cells. Ursolic acid, oleanolic acid, betulinic acid and their derivatives have been the focus of recent research on triterpenoids. Inhibition of HIV-1 protease has been observed *in vitro* with these triterpenes. One of the many betulinic acid derivatives that were synthesized in a structure-activity relationship study, RPR103611, was chosen for additional mechanistic research. Numerous important steps of the HIV life cycle have been inhibited by flavonoids. Viral protease has been inhibited by taxifolin which is a flavanone with an OH group at position C-3′. Aromadendrin does not inhibit viral protease or RT but can inhibit interactions between CD4 and gp120, is more definite in its antiviral activity and is a flavanone that lacks an OH group at position C-3′ (Mahmood et al., 1997). The (-)-epigallocatechin 3-*O*-gallate has demonstrated activity against HIV-1 (De Clercq, 1995) such as a damaging effect of virus particles and post-adsorption entry and inhibition of RT and viral protease (Yamaguchi et al., 2002). Flavonoids are recognized as inhibitors of several enzymes including RT which is crucial for HIV replication (Kitamura et al., 1998) viral protease (Xu et al., 2000) and integrase (Kim et al., 1998).

### Reverse Transcription

The viral reproductive cycle begins when the enzymes within the nucleoprotein complex are activated after the virus enters the cell. The viral RT transcribes the RNA genome of HIV into a double-stranded DNA form after the nucleoprotein core of the virus is disrupted. RNA reverse transcription to DNA, degradation of RNA template by RN-ase H and duplication of the remaining DNA strand are the three consecutive functions that are controlled by the HIV-1- RT. The 4-propyldipyranocoumarins or calanolides are natural products that have been considered to be potential anti-HIV agents. (+)-Calanolide A (Chan and Kim, 1998) is significantly more active on inhibition of HIV-1 RT when compared to cordatolides A (Jiang et al., 2002) and B (Lüscher-Mattli, 2000). Currently, to evaluate its pharmacokinetics and safety, (+)-calanolide A is being tested in clinical trials for both HIV-infected and healthy volunteers (Creagh et al., 2001).

### Integration

Besides protease and RT, integrase is the only enzyme that is encoded by HIV-1. Strand transfer and 3′-processing are the two steps for integration of viral DNA into host DNA and are catalyzed by integrase. This enzyme first cleaves the last two nucleotides from each 3′-end of the linear viral DNA. Then the nucleophilic attack of these 3′-ends on host chromosomal DNA are involved in the subsequent DNA strand transfer reaction. The l-chicoric acid was the most active inhibitor of HIV integrase out of all the bis-catechols tested. Recently, due to its compelling anti-HIV potential, MAP30 received a lot of attention and is a plant protein of 30 kDa extracted from Momordica charantia L., (Lee-Huang et al., 1995; Sun et al., 2001). Moreover, this plant protein toxicity is definite to HIV-infected cells, does not exhibit opposing effects on normal cells and is an inhibitor of HIV-1 integrase by rendering the HIV LTR an unsuitable substrate for HIV integrase as well as DNA gyrase.

### Transcription

Until levels of its trans-activator protein Tat rise, HIV-1 DNA transcription is low after integration of viral DNA into the host genome. By binding to the transactivation response element (TAR) at the 5′-end of the viral mRNA, Tat, an 86-amino acid polypeptide, can increase the capacity of host RNA polymerase (Ptak, 2002). The transcriptional activity of HIV-1 long terminal repeat (LTR) can be stimulated by the Tat protein after being released from HIV-1-infected cells and has entered new cells in an active form (Mann and Frankel, 1991). Between positions 133 and 104 of the HIV promoter, tannic acid has been observed to have found a putative tannic acid-responsive element and suppress 12-*O*-tetradecanoylphorbol 13-acetate (TPA) – inducing HIV promoter activity (Uchiumi et al., 1996). Furthermore, a number of natural substances related to tannins were assessed for their HIV promoter repression effects (Uchiumi et al., 2003). Chalcones, 3-phenylcoumarins and isoflavones have all successfully repressed TPA-induced HIV promoter activity in a structure-activity relationship study.

## Conclusion

Although, antiretroviral drugs can bring about the repression of the serum load of the virus to undetectable levels, economical, commercial, and political barriers have limited their accessibility to a good part of the population suffering from the diseases. Natural products, particularly those in traditional medicine have supplied a basis of new drug candidates for many diseases including HIV and other neurocognitive disorders. The number of compounds exhibiting anti-HIV activity isolated from plants is increasing gradually and due to their explicit activity, there are several natural products have been used as primary compounds. Since the resistance of HIV-1 to the antiretroviral drugs has been increasing along with the necessity of agents that are not as costly and toxic as the ones being used now, new treatments with these natural products are needed. The basic challenge that still persists is to develop viral replication-targeted therapy using novel anti-HIV compounds with new mode of action, accepted toxicity, and less resistance profile. Against this backdrop, the World Health Organization (WHO) suggested the need to evaluate ethno-medicines for the management of HIV/AIDS. Accordingly, there is need to evaluate traditional medicine, particularly medicinal plants and other natural products that may yield effective and affordable therapeutic agents (World Health Organization [WHO], 2002; Jaco et al., 2004; Hedimbi and Chinsembu, 2012). In this context, the above mentioned results demonstrate that several plants, the majority of which are usually utilized for the treatment of various illnesses, are active against HIV. Since they are the indicators for the decision making at various levels and the finding of new lead substances, these reports are invaluable. The preparation, cytotoxicity, or selectivity profile of an antiviral substance should also be carefully assessed before any further attention is given to it. However, in repressing HIV progression and replication it should be stressed that several natural products primarily extracted from plants have demonstrated to be effective. HIV/AIDS being an exceptional epidemic, demands an exceptional approach and that forms very much our current and future research.

## Conflict of Interest Statement

The authors declare that the research was conducted in the absence of any commercial or financial relationships that could be construed as a potential conflict of interest.
